# Chronic kidney disease patient safety in hemodialysis: evidence of scale validity and reliability

**DOI:** 10.1590/0034-7167-2024-0327

**Published:** 2025-08-08

**Authors:** Leticia Lima Aguiar, Marina Guerra Martins, Renan Alves Silva, Thereza Maria Magalhães Moreira, Paulo César de Almeida, Joselany Áfio Caetano

**Affiliations:** I Universidade Federal do Ceará. Fortaleza, Ceará, Brazil; II Universidade Federal de Campina Grande. Cajazeiras, Paraíba, Brazil; III Universidade Estadual do Ceará. Fortaleza, Ceará, Brazil

**Keywords:** Patient Safety, Hospital Hemodialysis Units, Nephrology Nursing, Renal Dialysis, Validation Studies., Seguridad del Paciente, Unidades Hospitalarias de Hemodiálisis, Enfermería de Nefrología, Diálisis Renal, Estudios de Validación.

## Abstract

**Objectives:**

to analyze evidence of the validity and reliability of the Chronic Kidney Disease Patient Safety Assessment Scale in Hemodialysis Sessions (EASPRCH).

**Methods:**

a methodological study with an analysis of validity evidence (content and construct) and reliability (temporal stability, internal consistency, and parameterization). A total of 11 judges and 240 patients from three hemodialysis clinics participated.

**Results:**

the EASPRCH consists of 15 items distributed across three domains: (1) Strategic Procedures for Error Prevention (6 items), (2) Organizational Processes in Health Management (5 items), and (3) Care Practices for Infection Control (4 items), with scoring available both by domain and overall. The scale has a maximum score of 45 points and a cutoff point (adequate/inadequate) of 34. It achieved a Cronbach’s alpha of 0.78 and explained 72.41% of the total variance.

**Conclusions:**

the scale provides evidence of weaknesses or strengths related to specific indicators, which may or may not impact patient safety in hemodialysis care.

## RESUMEN

**Objetivos:**

analizar evidencias de validez y confiabilidad de la Escala de Evaluación de la Seguridad del Paciente Renal Crónico en Sesión de Hemodiálisis (EASPRCH).

**Métodos:**

estudio metodológico con análisis de evidencias de validez (contenido y constructo) y confiabilidad (estabilidad temporal, consistencia interna y parametrización). Participaron 11 jueces y 240 pacientes de tres clínicas de hemodiálisis.

**Resultados:**

la EASPRCH consta de 15 ítems distribuidos en tres dominios: (1) Procedimientos Estratégicos para la Prevención de Errores (6 ítems), (2) Procesos Organizacionales de Gestión en Salud (5 ítems) y (3) Prácticas Asistenciales para el Control de Infecciones (4 ítems), con espacio para puntuación por dominio y global. Presenta una puntuación máxima de 45 puntos y un punto de corte (adecuado/no adecuado) de 34. Alcanzó un alfa de Cronbach de 0,78 y explicó el 72,41% de la varianza total.

**Conclusiones:**

la escala puede evidenciar fragilidades o potencialidades relacionadas con determinados indicadores, que pueden o no reflejarse en la seguridad de la atención al paciente en hemodiálisis.

## INTRODUCTION

In recent decades, patient safety has been a global concern, with significant challenges still present in ensuring high-quality and safe healthcare. Various strategies have been implemented to mitigate harm resulting from inadequate practices in healthcare services. However, despite these efforts, a desirable threshold of safety has not yet been achieved, as multiple factors influence the healthcare work process^(1)^.

This issue is particularly relevant in the care of individuals with chronic kidney disease (CKD) undergoing hemodialysis, given the high prevalence of the disease and the risk of incidents that may lead to adverse events. These events are commonly associated with care processes, inadequate communication between sectors, frequent handling of medical technology, administration of high-risk medications, fistula puncture management, catheter handling, assessment of patients’ hemodynamic conditions, and the risk of falls^(2)^.

Studies indicate that between 73% and 98% of patients experience at least one adverse event during dialysis treatment, with a prevalence ranging from 10% to 27% when considering the total number of hemodialysis sessions^(3-5)^. Therefore, strategies must be implemented to minimize risks in hemodialysis care, including individual and team training, simulations, the development of protocols, guidelines, checklists, and the use of information technology^(6,7)^.

A literature review on patient safety in hemodialysis highlights studies that focus on identifying adverse events and the main complications patients experience during treatment^(8,9)^. Furthermore, three safety checklists specific to hemodialysis have been identified, with only one, the *Hemo Pause Checklist*, having been adapted into Portuguese^(10)^. The other two are European checklists^(11,12)^. These three checklists share between 70% and 80% of their items and cover three phases of safety checks: pre-session, session initiation, and post-session.

Some limitations have been identified in these checklists, including a lack of coverage of essential patient safety aspects such as communication, medication administration, hand hygiene, proper puncture handling, and fall prevention. Additionally, these checklists do not provide clear outcomes regarding how patient safety will be assessed, as they function solely as verification tools.

To develop instruments capable of assessing safety in hemodialysis units and identifying noncompliance with legislation and safety standards, the creation and validation of the Chronic Kidney Disease Patient Safety Assessment Scale in Hemodialysis Sessions (EASPRCH in Portuguese) was initiated^(13)^. This scale was initially divided into six domains: correct patient identification, effective communication, management of high-risk medications, infection risk, fall risk, and proper procedures at intervention sites. The EASPRCH was validated with an intraclass correlation coefficient of 0.98 and a judge agreement rate of 0.96^(14)^.

This study aims to analyze evidence of the validity and reliability of the EASPRCH. The research question posed is: Is the EASPRCH a reliable and valid instrument for use in hemodialysis units?

Ensuring the effectiveness of treatment and the safety of CKD patients during hemodialysis sessions is crucial. In this context, implementing processes that reduce the occurrence of adverse events not only benefits the patient but also improves healthcare services by reducing staff workload, minimizing the consumption of resources needed to address complications, and ultimately decreasing the incidence of medical issues.

## OBJECTIVES

To analyze evidence of the validity and reliability of the Chronic Kidney Disease Patient Safety Assessment Scale in Hemodialysis Sessions.

## METHODS

### Ethical Aspects

The study was approved by the Research Ethics Committee of the affiliated university. Written informed consent form (ICF) was obtained from all individuals involved in the study.

### Study Design, Period, and Location

This is a methodological study conducted in accordance with the *Strengthening the Reporting of Observational Studies in Epidemiology* (STROBE) recommendations^(15)^ and carried out in two phases: (1) development of the scale and determination of content validity evidence, and (2) determination of evidence based on internal structure (pilot testing, construct validity, temporal stability, equivalence, internal consistency, and parameterization). The stages of this study were conducted from January 2019 to May 2020.

### Study Protocol

The development of the instrument, content validity assessment with researchers in the field of patient safety in hemodialysis, and semantic analysis with clinical practice professionals were conducted in a previous study. In that study, the instrument initially contained 57 items divided into six dimensions: correct patient identification, effective communication, high-risk medications, correct procedures and intervention sites, infection risk, and fall risk^(16)^.

The need to review and incorporate new items into the instrument was identified due to updates in clinical protocols and the expansion of the literature on patient safety in hemodialysis. Consequently, a review of the construct-related concepts was conducted to ensure clarity and comprehensibility for professionals. After these modifications, the scale was submitted to a new panel of specialists with expertise in patient safety, hemodialysis, and/or instrument validation.

In this study, experts were required to meet the following criteria: having at least one publication in the areas of patient safety, hemodialysis, and/or instrument validation; possessing specialized knowledge or skills in these fields; having at least 10 years of professional experience; and holding at least a specialist degree. Initially, the evaluation was conducted individually and online, with 11 judges. To assess the relevance/representativeness of the items, the judges could choose from the following responses: 1 = not relevant or not representative, 2 = item requires major revision to be representative, 3 = item requires minor revision to be representative, or 4 = relevant or representative item. The criteria of comprehensiveness, clarity, and pertinence were also assessed using the same scale^(17)^. A minimum Content Validity Index (CVI) of 0.8 was considered acceptable for the evaluation of each individual item, while a CVI of 0.90 was required for the overall scale assessment.

Following this online phase, a discussion session was conducted to analyze the CVI results and discuss the feedback and suggestions provided. However, only three specialist judges were able to participate in the two in-person meetings.

In the next stage, a pilot test was conducted with 50 patients with CKD undergoing hemodialysis at three specialized clinics. The patient sample was selected using proportional stratified probability sampling. The three HD clinics were located in different regions of Fortaleza, Ceará, Brazil, and collectively served 547 patients in treatment rooms accommodating four to twelve patients, with one to three nursing technicians present per shift.

The instrument was developed for observational application, allowing the researcher to collect data during the hemodialysis session, including the reuse process and water records. The scale scores varied according to the quality of care provided to each patient, with ratings ranging from 0 - Not Applicable (NA) to 3 - Compliant (C). It is recommended that one instrument be used per patient, evaluating all care provided throughout the hemodialysis session. The application time corresponds to an entire session, from room preparation to the patient’s discharge, including post-discharge care.

After the pilot test, the scale was applied to 240 patients (77 from Clinic A, 44 from Clinic B, and 119 from Clinic C), maintaining the proportional distribution of patients from each clinic. A significance level of α = 0.05 and a power of 80% were adopted. The inclusion criteria for the study were: being an adult CKD patient, being at least 18 years old, and undergoing hemodialysis treatment at one of the selected clinics for at least one month. Patients who did not complete the hemodialysis session due to clinical instability were excluded from the study.

### Analysis of Results and Statistics

Among the tests used in the factor analysis (FA), the following analyses were performed: means and standard deviations of the EASPRCH items, correlations, Cronbach’s alpha, communalities, total variance explained, Scree plot, and principal component matrix. The adequacy of the Exploratory Factor Analysis (EFA) was tested to determine whether Bartlett’s sphericity test was significant at the 0.05 level and whether the Kaiser-Meyer-Olkin (KMO) index was greater than 0.70^(18)^.

Regarding correlations, items with a correlation below 0.30 were considered potential candidates for exclusion. For the evaluation of communality, a value greater than 0.40 was considered satisfactory. The number of factors in the EASPRCH was also confirmed using the Scree test, total variance explained, and eigenvalues greater than 1. A rotated component matrix was generated, and in this study, a minimum factor loading of 0.40 was adopted for an item to be considered a useful representative of the factor.

In summary, to test construct validity, determine factors, and retain items, the following criteria were applied:

1. Items with low means, below 0.5;
2. Low variability, with a standard deviation equal to 0.00;
3. Low correlation between items, below 0.30;
4. Items with communality and factor loading below 0.40.

The internal consistency of the EASPRCH was analyzed using Cronbach’s alpha, with a value of 0.7 considered acceptable. Equivalence and temporal stability were also assessed using the test-retest method, conducted by the same researcher after 14 days or during the following hemodialysis session. The sample size calculation based on the Kappa coefficient was 42, and therefore, Kappa values greater than 0.40 were adopted^(19)^.

To define the levels of the EASPRCH, the Receiver Operating Characteristic (ROC) curve was used^(20)^, with the optimal cutoff point determined as the value that achieved the highest sensitivity and specificity for the scale.

In this study, a significance level of 5% was adopted for all statistical tests. The collected data were stored in an electronic spreadsheet and imported for analysis using SPSS 19.

## RESULTS

The initial version of the EASPRCH contained 57 items distributed across six dimensions. This version was evaluated virtually by eleven specialists in the fields of patient safety, hemodialysis, and instrument validation. The suggestions provided by these specialists were consolidated in an in-person meeting with three of them, resulting in the exclusion of 12 items, the inclusion of three new ones, and the revision of 36 items. With these modifications, the scale was reduced to 48 items.

In the next phase, a pilot test was conducted, leading to changes in 16 items. After the pilot test, the modified scale was applied to 240 patients with CKD undergoing hemodialysis in three clinics located in Fortaleza, Ceará, Brazil.

In the final phase, evidence of the scale’s construct validity was analyzed, resulting in the exclusion of 24 items. Additionally, five items were removed based on the correlation matrix, and four others were eliminated after assessing internal consistency. After applying the scale to a group of 50 patients, a semantic analysis was conducted, during which assistant researchers participated in a discussion session with the principal researcher. During this meeting, operational redefinitions were made to improve the comprehension of the EASPRCH.

The CVI of the modified version of the EASPRCH ranged from 0.56 to 1.0, with full agreement on the criteria of clarity and relevance. Although adequate internal consistency was not achieved for three of the 15 items on the scale, they were retained because they represent fundamental actions within the context of patient safety in HD, as recommended by both national and international literature.

The remaining 12 items demonstrated strong factorial relationships. FA showed that the presence of three components with eigenvalues greater than 1.00 in the EASPRCH explained 72.41% of patient safety practices in CKD hemodialysis. The first factor accounted for 35.28% of the variance, the second for 26.42%, and the third for 10.70%. Thus, the EASPRCH achieved more than 70% of the explained variance (Table 1).

**Table 1:** Rotated component matrix of the EASPRCH, Fortaleza, Ceará, Brazil, 2021

Items	Components		
	1	2	3
1	.984		
2	.993		
3		.644	
4	.993		
5		.977	
6	.993		
7		.934	
8			.596
9	.420		
10			.742
11			.696
12		.743	
13		.977	
14	.993		
15		.414	.440

*Extraction Method: Principal Component Analysis; Rotation Method: Varimax with Kaiser Normalization.*

The KMO value was 0.5. The sphericity test indicated statistical significance (p < 0.001), demonstrating sufficient correlation among the items for FA. Items 1, 2, 4, 6, 9, and 14 formed Factor 1 (Cronbach’s alpha = 0.93); items 3, 5, 7, 12, and 13 comprised Factor 2 (Cronbach’s alpha = 0.86); and items 8, 10, 11, and 15 formed Factor 3 (Cronbach’s alpha = 0.49). The total Cronbach’s alpha for the scale was 0.78.

Furthermore, in the test-retest, the Kappa value ranged from 0.24 to 1.00, with four items presenting values below the recommended threshold. In terms of reproducibility, ten items showed negative Kappa values, ranging from 0.089 to 1.00 (Table 2).

**Table 2:** Inter-test and Inter-observer Agreement Analysis, Fortaleza, Ceará, Brazil, 2021

	Inter-Test Kappa Coefficient	*p* ^*^	Inter-Observer Kappa Coefficient	*p* †
Item 1	1	<0.001	-0.065	0.606
Item 2	1	<0.001	-0.074	0.557
Item 3	1	1.00	1.00	1.00
Item 4	0.901	<0.001	-0.043	0.723
Item 5	0.851	<0.001	0.730	<0.001
Item 6	1	<0.001	0.075	0.345
Item 7	1	1.00	1.00	1.00
Item 8	0.240	0.012	0.053	0.386
Item 9	0.311	0.002	0.086	0.317
Item 10	0.325	0.014	0.387	0.007
Item 11	0.633	<0.001	0.089	0.391
Item 12	0.604	<0.001	-0.089	0.534
Item 13	0.883	<0.001	0.729	<0.001
Item 14	1	<0.001	0.264	<0.001
Item 15	0.414	0.001	-0.075	0.248

p^*^ = p-value for inter-test agreement; p^†^ = p-value for inter-observer agreement.

In the parameterization, the cutoff score was defined as 34, as this value was estimated using the ROC curve based on the optimal combination of sensitivity (100%) and specificity (80%). Thus, it was determined that scores above 34 points indicate that the institution has stronger potential indicators for practices that ensure patient safety in hemodialysis, while scores below 34 points suggest greater vulnerabilities. This identification can support the implementation of measures and interventions aimed at improving the most problematic indicators identified through the scale.

The modified version of the EASPRCH consists of 15 items, organized on a Likert scale ranging from zero to three points, with a total maximum score of 45 points and a cutoff point for adequacy set at 34 points. The scale is structured into three dimensions: (1) Strategic Procedures for Error Prevention (6 items), (2) Organizational Processes in Health Management (5 items), and (3) Care Practices for Infection Control (4 items) (Table 3).

**Table 3:** Modified version of the Chronic Kidney Disease Patient Safety Assessment Scale in Hemodialysis Clinics (EASPRCHP), Fortaleza, Ceará, Brazil, 2021

Procedimentos Estratégicos para Prevenção de Erros				
	NA	NC	PC	C
1. Legible identification of the dialyzer with the patient's full name, date of birth, and date of the first use of the system; and dialysis lines with the patient’s full name.	0	1	2	3
2. Identification of the hemodialysis system box using at least two identifiers, such as the patient’s full name, date of birth, medical record number, differentiation by shifts, or serology status.	0	1	2	3
3. Storage of processed dialyzers in rigid, easily sanitized, individualized containers with a closed lid.	0	1	2	3
4. Performing reagent testing of the hemodialysis system before each session to certify system disinfection.	0	1	2	3
5. Recording the internal fiber volume measurement of the dialyzer (priming) before first use and after each reprocessing, indicating the number of uses, as well as the date and signature of the professional responsible for reprocessing.	0	1	2	3
6. Changing gloves for each new procedure (such as dressing changes and handling of the hemodialysis system) by healthcare professionals.	0	1	2	3
**Partial Score:**				
Non-Conformity Aspects:				
**Organizational Processes in Health Management**				
	NA	NC	PC	C
7. Use of labels to record intravenous medication administration with the medication name, dose, date, time of dilution/aspiration, name of the responsible professional, and the patient’s full name.	0	1	2	3
8. Storage of potentially hazardous medications in an exclusive, identified, restricted-access location with opening control.	0	1	2	3
9. Use of a disposable isolator to measure arterial and venous pressure on the machine.	0	1	2	3
10. Biannual record of potable water reservoir cleaning, signed by the responsible professional.	0	1	2	3
11. Presence of a nursing station near the hemodialysis room with easy access, visibility of all patients, and availability of necessary material resources for assistance.	0	1	2	3
**Partial Score:**				
Non-Conformity Aspects:				
**Care Practices for Infection Control**				
	NA	NC	PC	C
12. Removal of accessories and use of PPE by healthcare professionals.	0	1	2	3
13. Hand hygiene by healthcare professionals before touching the patient, before performing aseptic procedures, after exposure to body fluids, and after contact with surfaces near the patient.	0	1	2	3
14. Dressing of catheters and/or fistulas using an aseptic technique.	0	1	2	3
15. Cleaning and drying of the floor after each hemodialysis session and whenever necessary.	0	1	2	3
**Partial Score:**				
Non-Conformity Aspects:				
Total Score:				

*Notes: 0 - (NA) Not Applicable: The entire content of the item does not apply to the reality being assessed or cannot be observed.; 1 - (NC) Non-Compliant: The entire content of the item is not in compliance with the reality being assessed.; 2 - (PC) Partially Compliant: Only part of the item’s content is consistent with the reality being assessed; 3 - (C) Compliant: The entire content of the item is in compliance with the reality being assessed.*

## DISCUSSION

During the validation of the internal structure of the EASPRCH, some items were excluded due to a standard deviation of zero. These invariabilities are attributed to the basic requirements necessary for the operation of dialysis services and other patient safety regulations^(13,21)^.

Regarding internal consistency, the EASPRCH presented Cronbach’s alpha values similar to those of other instruments related to patient safety, such as the Scale of Predisposition to the Occurrence of Adverse Events (EPEA) (Cronbach’s alpha = 0.74 for the Structure dimension and 0.93 for the Process dimension)^(22)^, the Safety Attitudes Questionnaire (SAQ) (Cronbach’s alpha = 0.89), and the Hospital Survey on Patient Safety Culture (HSOPSC) (Cronbach’s alpha = 0.91)^(23,24)^.

The modified version of the EASPRCH, with 15 items, achieved 72.41% of explained variance, aligning with other instruments, such as the Scale of Adverse Events Associated with Nursing Practices, which explained 70.79% of the variance with eleven factors^(25)^.

The principal component matrix, with Varimax rotation, showed a better division of the scale into three factors, namely: (1) Strategic Procedures for Error Prevention, with items related to identification, disinfection, functionality, and proper storage of supplies used during hemodialysis sessions, as well as the use of personal protective equipment (PPE); (2) Organizational Processes in Health Management, adjusted with items such as medication safety, use of disposable isolators, standards and routines for water treatment, and functional physical infrastructure; and (3) Care Practices for Infection Control, including standards and routines regarding the use of PPE, hand hygiene, aseptic techniques for wound care, and environmental cleaning.

The items that comprise the scale dimensions demonstrate that adverse events result from the combination of multiple factors, including individual patient characteristics, the conditions of healthcare professionals, and institutional variables. In the hemodialysis sector, this complexity is intensified by the use of advanced equipment, the need for rigorous water treatment, high patient turnover, and the administration of potentially hazardous medications. Additionally, the risk of infection is high, requiring meticulous care and well-defined safety protocols to minimize complications. These combined factors make the hemodialysis environment particularly susceptible to adverse events, highlighting the importance of instruments that assess patient safety^(26)^.

The dimensions of the EASPRCH are associated with the six areas that the Renal Physicians Association (RPA) proposes for improving patient safety: hand hygiene; correct use of the dialyzer and dialysis solution; safe medication administration; adherence to protocols, standards, and routines; fall prevention; and care related to fistula puncture^(9)^.

According to a review study, the analysis of the items and their respective domains in the EASPRCH reveals that they align with predisposing, disabling, precipitating, and reinforcing factors related to the safety culture of CKD patients undergoing hemodialysis treatment^(27)^. A study conducted in Japan, for example, developed a system called *Error Taxonomy* and concluded that more than 70% of hemodialysis incidents were associated with problems or complications involving the dialyzer, the circuit, or medication. These findings reinforce the relevance of the first two dimensions of the scale, which directly address these crucial aspects of patient safety^(28)^.

In Mexico, it was found that the most prevalent adverse events in patients undergoing hemodialysis were related to vascular access infections, reinforcing the importance of the third dimension of the EASPRCH^(29)^.

Additionally, when analyzing the temporal stability of the scale, it was observed that the content of the items with Kappa values below the recommended threshold was related to behavioral factors of healthcare professionals. This finding in the present study justifies the temporal instability of these items and the possibility of external interference. Studies indicate that lower Kappa values are associated with researchers’ understanding or the time interval between measurements, as an excessively long interval may lead to changes in the subject’s health status, modifying the evaluation criteria^(30-32)^.

Finally, in the parameterization, the cutoff point of the EASPRCH was defined using the ROC curve. It is assumed that when the scale presents high scores (>34), the occurrence of incidents will be reduced, whereas low scores on the scale (<34) may predict a higher incidence of hemodialysis-related incidents.

The analysis of the scale scores, as well as its individual items, can contribute to risk management in the occurrence of incidents during hemodialysis sessions, given that this is a dynamic and complex environment. Risk management involves a systematic process of identifying, analyzing, and controlling actual and potential risks, considering the implications associated with the available resources. This process goes beyond merely identifying errors, highlighting the importance of proposing proactive solutions to reduce and mitigate failures, continuously promoting patient safety^(33)^.

Hemodialysis patients are more prone to developing metabolic disorders, coagulation abnormalities, and bloodstream infections. These issues directly impact the work of healthcare professionals. The importance of continuous care for these individuals is emphasized, with organization and planning aimed at preventing and treating complications associated with the procedure^(34,35)^. Therefore, the application and analysis of the scores and information collected by the EASPRCH will provide data to guide and monitor functional and organizational weaknesses, assisting in better professional training and improving the work process of the nursing team.

### Study limitations

Certain limitations need to be acknowledged, such as the restricted scope of data collection. Although conducted with a significant sample of the population, data collection took place in only three hemodialysis clinics located in the same state and city, within relatively similar settings. Additionally, it is suggested that new data collection be conducted to test the reproducibility of the scale. However, this time, only one instrument should be applied per session to allow for reassessment and verification of the scale’s data replication capacity by different researchers. It is also recommended that the EASPRCH be adapted for acute renal patients undergoing dialysis in hospitals, a setting that remains understudied and presents multiple variations in patients’ clinical conditions, which may compromise safety.

### Contributions to the Field

This article reinforces the importance of instruments that guide and promote patient safety in hemodialysis clinics, serving as a starting point for the development of additional instruments tailored to the Brazilian context, integrating patient safety with the hemodialysis procedure. Furthermore, it highlights the need for studies that provide resources for the practical implementation of Patient Safety Units, as well as for the identification of incidents and adverse events in hemodialysis.

## CONCLUSIONS

The modified version of the EASPRCH is made available to readers, with evidence of construct validity, reliability, and stability, including a cutoff point that enables the identification of weaknesses or strengths related to specific indicators, which may or may not impact patient safety in hemodialysis care. This allows for the strategic direction of measures and interventions that enhance safe practices in patient care during hemodialysis.

## Data Availability

Not applicable.
