# Supplementary material for: Chronic kidney disease patient safety in hemodialysis: evidence of scale validity and reliability
Source: Rev Bras Enferm. 2025 Aug 8;78(3):e20240327. doi: 10.1590/0034-7167-2024-0327 (PMC12334197; doi:10.1590/0034-7167-2024-0327)
Supplement: Translation

# Translation: Segurança do paciente renal crônico em hemodiálise: evidências de validade e confiabilidade da escala

**DOI:** 10.1590/0034-7167-2024-0327pt

**Authors:** Leticia Lima Aguiar, Marina Guerra Martins, Renan Alves Silva, Thereza Maria Magalhães Moreira, Paulo César de Almeida, Joselany Áfio Caetano

**Affiliations:** I Universidade Federal do Ceará. Fortaleza, Ceará, Brasil; II Universidade Federal de Campina Grande. Cajazeiras, Paraíba, Brasil; III Universidade Estadual do Ceará. Fortaleza, Ceará, Brasil

**Keywords:** Segurança do Paciente, Unidades Hospitalares de Hemodiálise, Enfermagem em Nefrologia, Diálise Renal, Estudos de Validação.

## Abstract

**Objetivos:**

analisar evidências de validade e confiabilidade da Escala de Avaliação da Segurança do Paciente Renal Crônico em Sessão de Hemodiálise (EASPRCH).

**Métodos:**

estudo metodológico, com análise de evidências de validade (conteúdo e construto) e confiabilidade (estabilidade temporal, consistência interna e parametrização). Participaram 11 juízes e 240 pacientes de três clínicas de hemodiálise.

**Resultados:**

a EASPRCH possui 15 itens e três domínios: (1) Procedimentos Estratégicos para Prevenção de Erros (6 itens), (2) Processos Organizacionais de Gestão em Saúde (5 itens) e (3) Práticas Assistenciais para Controle de Infecção (4 itens), com espaço para pontuação por domínio e global. Apresenta pontuação máxima de 45 pontos e ponto de corte (adequado/não adequado) de 34. Alcançou alfa de Cronbach de 0,78 e 72,41% de variância total explicada.

**Conclusões:**

a escala pode demonstrar evidências de fragilidades ou potencialidades relacionadas a determinados indicadores, que podem refletir ou não na segurança do atendimento ao paciente em hemodiálise.

## INTRODUÇÃO

Nas últimas décadas, a segurança do paciente tem sido uma preocupação mundial, cujos desafios ainda são imensos para promover uma assistência de qualidade e segura. Assim, diversas estratégias vêm sendo instituídas para mitigar os danos oriundos de práticas inadequadas nos serviços assistenciais. Entretanto, apesar dos esforços realizados, ainda não se alcançou um limiar desejável de segurança, pois vários fatores estão envolvidos no processo de trabalho em saúde^(1)^.

Essa temática é de grande relevância, especialmente no que se refere à atenção à pessoa com doença renal crônica (DRC) em unidades de hemodiálise, dada a prevalência da doença e o risco de incidentes que podem resultar em eventos adversos. Esses eventos, comumente, estão relacionados ao processo de assistência, à comunicação inadequada entre setores, ao manuseio constante de tecnologias, à administração de medicamentos potencialmente perigosos, aos cuidados com a punção da fístula, ao manuseio do cateter, à análise das condições hemodinâmicas do paciente e ao risco de queda^(2)^.

Estudos mostram que 73% a 98% dos pacientes sofrem pelo menos um evento adverso durante o tratamento dialítico e que a prevalência desses eventos, considerando o total de sessões de hemodiálise, varia de 10% a 27%^(3-5)^. No entanto, estratégias devem ser implementadas para minimizar os riscos na assistência em unidades de hemodiálise, como treinamento individual e em equipe, simulações, desenvolvimento de protocolos, diretrizes, listas de verificação e uso da tecnologia da informação^(6,7)^.

Em busca na literatura sobre segurança do paciente em hemodiálise, os estudos enfatizam a verificação dos eventos adversos e das principais intercorrências apresentadas pelos pacientes durante o tratamento^(8,9)^. Além disso, foram identificadas três listas de verificação de segurança aplicadas à hemodiálise, sendo apenas uma delas, a *Hemo Pause Checklist*, adaptada para o português^(10)^. As outras duas listas são europeias^(11,12)^. As três listas compartilham entre 70% e 80% dos itens e abrangem três fases de verificações de segurança: pré-sessão, início da sessão e pós-sessão.

Algumas limitações nas listas de verificação foram percebidas, como a não abordagem de aspectos importantes na segurança do paciente, tais como: comunicação, administração de medicamentos, higienização das mãos, manuseio correto da punção e prevenção de quedas. Além disso, não apresentam resultados claros sobre como a segurança do paciente será avaliada, pois são apenas listas de verificação.

Portanto, para desenvolver instrumentos que avaliem a segurança nas unidades de hemodiálise e identifiquem inconformidades com a legislação e os padrões de segurança, iniciou-se a criação e a validação da Escala de Avaliação da Segurança do Paciente Renal Crônico em Sessão de Hemodiálise (EASPRCH)^(13)^. Essa escala foi inicialmente dividida em seis domínios: identificação correta do paciente; comunicação efetiva; cuidados com medicamentos potencialmente perigosos; risco de infecção; risco de queda; e procedimentos corretos nos locais de intervenção. A EASPRCH foi validada com coeficiente de correlação intraclasse de 0,98 e concordância entre os juízes de 0,96^(14)^.

Este estudo pretende analisar evidências de validade e confiabilidade da EASPRCH. A pergunta de pesquisa é: a EASPRCH. é confiável e válida para ser utilizada nas unidades de hemodiálise?

Ressalta-se que é crucial assegurar a eficácia do tratamento e a segurança do paciente renal crônico em sessão de hemodiálise. Nesse contexto, a implementação de processos que possibilitem a redução da ocorrência de eventos adversos não só traz benefícios para o paciente, mas também para o serviço de saúde, com a redução da carga de trabalho da equipe, menor consumo de insumos para o tratamento de intercorrências e diminuição de complicações.

## OBJETIVOS

Analisar evidências de validade e confiabilidade da Escala de Avaliação da Segurança do Paciente Renal Crônico em Sessão de Hemodiálise (EASPRCH).

## MÉTODOS

### Aspectos éticos

A pesquisa foi aprovada pelo Comitê de Ética em Pesquisa da universidade à qual está vinculada. O consentimento livre e esclarecido foi obtido, por escrito, de todos os indivíduos envolvidos no estudo.

### Desenho, período e local do estudo

Trata-se de um estudo metodológico, descrito a partir das recomendações *Strengthening the Reporting of Observational Studies in Epidemiology* (STROBE)^(15)^, realizado em duas etapas: (1) construção da escala e determinação da evidência de validade baseada no conteúdo e (2) determinação da evidência baseada na estrutura interna (teste piloto, validade de construto, estabilidade temporal, equivalência, consistência interna e parametrização). As etapas que compõem este estudo foram realizadas de janeiro de 2019 a maio de 2020.

### Protocolo do estudo

A construção, a validade de conteúdo com pesquisadores na área de segurança do paciente em hemodiálise e a análise semântica com profissionais da prática clínica foram realizadas em um estudo anterior, no qual o instrumento continha 57 itens, divididos em seis dimensões: identificação correta do paciente, comunicação efetiva, medicamentos potencialmente perigosos, procedimentos e local de intervenção corretos, risco de infecção e risco de quedas^(16)^.

Reconheceu-se a necessidade de revisar e incorporar novos itens ao instrumento devido à atualização de protocolos clínicos e à ampliação da literatura sobre segurança do paciente em hemodiálise. Dessa forma, foi realizada uma revisão dos conceitos do construto relacionado à temática, assegurando que estivessem claros e compreensíveis para os profissionais. Após as modificações, a escala foi submetida a um novo comitê de especialistas com experiência em segurança do paciente, hemodiálise e/ou validação de instrumentos.

Nesta pesquisa, os experts possuíam as seguintes características obrigatórias: publicação em pelo menos uma das áreas (segurança do paciente, hemodiálise e/ou validação de instrumentos); corpo de conhecimento especializado ou habilidade nas áreas mencionadas; elevado tempo de graduação (pelo menos 10 anos); e, no mínimo, o título de especialista. Inicialmente, a avaliação foi realizada individualmente, de maneira online, com 11 juízes. Para avaliar a relevância/representatividade dos itens, os juízes puderam escolher entre as seguintes respostas: 1 = não relevante ou não representativo, 2 = item necessita de grande revisão para ser representativo, 3 = item necessita de pequena revisão para ser representativo, ou 4 = item relevante ou representativo. Os critérios de abrangência, clareza e pertinência também foram avaliados com a mesma escala^(17)^. Foi considerado um Índice de Validade de Conteúdo (IVC) mínimo de 0,8 como aceitável para a avaliação de cada item individualmente e de 0,90 para a avaliação geral da escala.

Após essa etapa online, seguiu-se com uma roda de conversa para analisar os IVC e discutir as colocações e sugestões enviadas; entretanto, apenas três juízes especialistas puderam participar dos dois encontros presenciais.

Na etapa seguinte, foi realizado um teste piloto com 50 pacientes com DRC em hemodiálise de três clínicas especializadas. A amostragem para a escolha dos pacientes foi probabilística estratificada proporcional. As três clínicas de hemodiálise estavam situadas em diferentes regiões de Fortaleza, Ceará, Brasil, e possuíam, juntas, 547 pacientes, atendidos em salas com quatro a doze pacientes e a presença de um a três técnicos de enfermagem nos três turnos.

O instrumento foi desenvolvido para aplicação observacional, permitindo que o pesquisador coletasse informações durante a sessão de hemodiálise, inclusive no processo de reuso e nos registros de água. Os escores da escala variam conforme a assistência prestada a cada paciente, com pontuações de 0 - Não se aplica (NA) a 3 - Conforme (C). Recomenda-se a utilização de um instrumento por paciente, avaliando toda a assistência prestada ao longo da sessão de hemodiálise. O tempo de aplicação corresponde a uma sessão completa, desde a preparação da sala até a saída do paciente, incluindo os cuidados realizados após sua saída.

Após o teste piloto, a escala foi aplicada a 240 pacientes (77 da clínica A, 44 da clínica B e 119 da clínica C), obedecendo à proporção de pacientes de cada clínica. Foram adotados os parâmetros de nível de significância α = 0,05 e poder de 80%. Os critérios de inclusão no estudo foram: ser paciente renal crônico adulto; ter idade igual ou superior a 18 anos; e realizar tratamento de hemodiálise nas clínicas selecionadas há pelo menos um mês. Foram excluídos do estudo pacientes que não completaram a sessão de hemodiálise por instabilidade clínica.

### Análise dos resultados e estatística

Dentre os testes utilizados na análise fatorial (AF), foram realizadas as seguintes análises: médias e desvio padrão dos itens da EASPRCH, correlações, alfa de Cronbach, comunalidades, variância total explicada, *Scree plot* e matriz de componentes principais. A adequação da Análise Fatorial Exploratória (AFE) foi testada com a finalidade de avaliar se o teste de esfericidade de Bartlett era significativo ao nível de 0,05 e se o índice Kaiser-Meyer-Olkin (KMO) era superior a 0,70^(18)^.

Com relação às correlações, os itens com correlação inferior a 0,30 foram considerados possíveis candidatos à exclusão. Para a avaliação da comunalidade, considerou-se satisfatório um valor superior a 0,40. O número de fatores da EASPRCH também foi confirmado pelo diagrama de declividade *Scree test*, pela variância total explicada e pelo autovalor maior que 1. Realizou-se a matriz de componentes rotacionada, adotando-se, neste estudo, uma carga fatorial mínima de 0,40 para que o item pudesse ser considerado um representante útil do fator.

Em resumo, para testar a validade de construto, a determinação de fatores e a retenção de itens, utilizaram-se os seguintes critérios:

1. Valores com médias baixas, menores que 0,5;
2. Baixa variabilidade, com desvio padrão igual a 0,00;
3. Baixa correlação entre os itens, inferiores a 0,30;
4. Itens com comunalidade e carga fatorial inferiores a 0,40.

A consistência interna da EASPRCH foi analisada pelo coeficiente alfa de Cronbach, considerando-se um valor de 0,7 como aceitável. Também foram avaliadas sua equivalência e estabilidade no tempo por meio do teste-reteste, realizado pelo mesmo pesquisador após 14 dias ou na sessão de hemodiálise seguinte. O cálculo amostral com base no coeficiente Kappa foi 42 e, assim, adotaram-se valores de Kappa superiores a 0,40^(19)^.

Para a definição dos níveis da EASPRCH, utilizou-se a Curva *Receiver Operating Characteristic* (ROC)^(20)^, sendo definido como ponto ideal aquele que apresentasse o máximo de sensibilidade e especificidade da escala.

Neste estudo, adotou-se um nível de significância de 5% para todos os testes estatísticos. Os dados coletados foram armazenados em uma planilha eletrônica e importados para análise no programa SPSS, v.19.

## RESULTADOS

A versão inicial da EASPRCH continha 57 itens distribuídos em seis dimensões. Essa versão foi avaliada virtualmente por onze especialistas nas áreas de segurança, hemodiálise e validação de instrumentos. As sugestões desses especialistas foram consolidadas em um encontro presencial com três deles, resultando na exclusão de 12 itens, na inclusão de três novos e na reformulação de 36 itens. Com essas modificações, a escala passou a conter 48 itens.

Na etapa seguinte, foi realizado um teste piloto, que levou a alterações em 16 itens. Após o piloto, a escala modificada foi aplicada a 240 pacientes com DRC em hemodiálise, de três clínicas localizadas em Fortaleza, Ceará, Brasil.

Na etapa final, foram analisadas as evidências de validade de construto da escala, resultando na exclusão de 24 itens. Além disso, cinco itens foram eliminados com base na matriz de correlações e outros quatro, na verificação da consistência interna. Após a aplicação da escala em um grupo de 50 pacientes, foi realizada uma análise semântica, durante a qual as pesquisadoras auxiliares participaram de uma roda de conversa com a pesquisadora principal. Nesse encontro, foram feitas redefinições operacionais, o que aumentou a compreensão da EASPRCH.

O IVC da versão modificada da EASPRCH variou de 0,56 a 1,0, com total concordância nos critérios de clareza e relevância. Embora a consistência interna adequada não tenha sido alcançada para três dos 15 itens da escala, optou-se por mantê-los, pois eles representam ações fundamentais no contexto da segurança do paciente em hemodiálise, conforme recomendado pela literatura nacional e internacional.

Os demais 12 itens apresentaram boa relação fatorial. A AF mostrou que a presença de três componentes com autovalor maior que 1,00 na EASPRCH explica 72,41% da prática de segurança do paciente renal crônico em hemodiálise. Nota-se que o primeiro fator é responsável por uma variância de 35,28%, o segundo por 26,42% e o terceiro por 10,70%. Assim, a EASPRCH atingiu mais de 70% de variância explicada (Tabela 1).

**Tabela 1:** Matriz das Componentes Rotacionadas da EASPRCH, Fortaleza, Ceará, Brasil, 2021

Itens	Componentes		
	1	2	3
1	,984		
2	,993		
3		,644	
4	,993		
5		,977	
6	,993		
7		,934	
8			,596
9	,420		
10			,742
11			,696
12		,743	
13		,977	
14	,993		
15		,414	,440

*Método de Extração: Análise de Componente Principal; Método de Rotação: Varimax com Normalização de Kaiser.*

O valor do KMO foi 0,5. O teste de esfericidade indicou significância estatística (p < 0,001), demonstrando correlação entre os itens suficiente para a AF. Os itens 1, 2, 4, 6, 9 e 14 formaram o fator 1 (alfa de Cronbach = 0,93); os itens 3, 5, 7, 12 e 13 compuseram o fator 2 (alfa de Cronbach = 0,86); e os itens 8, 10, 11 e 15 formaram o fator 3 (alfa de Cronbach = 0,49). O alfa de Cronbach total da escala foi de 0,78.

Ademais, no teste-reteste, o valor de Kappa variou de 0,24 a 1,00, com quatro itens apresentando valor inferior ao recomendado. Na reprodutibilidade, dez itens apresentaram Kappa negativo, variando de 0,089 a 1,00 (Tabela 2).

**Tabela 2:** Análise de Concordância Intertestes e Inter-observadores, Fortaleza, Ceará, Brasil, 2021

	Coeficiente de Kappa Inter Teste	*p* ^*^	Coeficiente de Kappa Inter Observadores	*p* †
Item 1	1	<0,001	-0,065	0,606
Item 2	1	<0,001	-0,074	0,557
Item 3	1	1,00	1,00	1,00
Item 4	0,901	<0,001	-0,043	0,723
Item 5	0,851	<0,001	0,730	<0,001
Item 6	1	<0,001	0,075	0,345
Item 7	1	1,00	1,00	1,00
Item 8	0,240	0,012	0,053	0,386
Item 9	0,311	0,002	0,086	0,317
Item 10	0,325	0,014	0,387	0,007
Item 11	0,633	<0,001	0,089	0,391
Item 12	0,604	<0,001	-0,089	0,534
Item 13	0,883	<0,001	0,729	<0,001
Item 14	1	<0,001	0,264	<0,001
Item 15	0,414	0,001	-0,075	0,248

* Valor de p Inter teste;

† valor de p Inter observadores (teste Kappa).

Na parametrização, o ponto de corte definido foi o escore 34, pois, por meio da Curva ROC, esse valor foi estimado com base na melhor combinação entre sensibilidade (100%) e especificidade (80%). Assim, constatou-se que escores superiores a 34 pontos indicam que a instituição possui indicadores de maior potencialidade para práticas que garantam a segurança do paciente em hemodiálise, enquanto escores inferiores a 34 pontos apontam maiores fragilidades. Essa identificação poderá subsidiar a implementação de medidas e intervenções voltadas para a melhoria dos indicadores mais problemáticos identificados a partir da escala.

A versão modificada da EASPRCH ficou com 15 itens, organizados em uma escala Likert de zero a três pontos, com pontuação máxima total de 45 pontos e ponto de corte adequado/não adequado em 34 pontos. A escala foi estruturada em três dimensões: (1) Procedimentos Estratégicos para Prevenção de Erros (6 itens), (2) Processos Organizacionais de Gestão em Saúde (5 itens) e (3) Práticas Assistenciais para Controle de Infecção (4 itens) (Tabela 3).

**Tabela 3:** Versão modificada da Escala de avaliação da segurança do paciente renal crônico em clínica de hemodiálise (EASPRCHP), Fortaleza, Ceará, Brasil, 2021

Procedimentos Estratégicos para Prevenção de Erros				
	NA	NC	PC	C
1 - Identificação legível do dialisador com nome completo do paciente, data de nascimento e data do primeiro uso do sistema; e linhas de diálise com nome completo do paciente.	0	1	2	3
2 - Identificação da caixa do sistema de hemodiálise, usando pelo menos dois identificadores, como: nome completo do paciente, data de nascimento, número do prontuário, diferenciação por turnos ou sorologias.	0	1	2	3
3 - Acondicionamento dos dialisadores processados em recipientes rígidos, de fácil higienização, individualizado e fechado com tampa.	0	1	2	3
4 - Realização de teste com reagente do sistema de hemodiálise antes de cada sessão, para certificação da desinfecção do sistema.	0	1	2	3
5 - Registros de medida do volume interno das fibras do dialisador (*priming*) antes do primeiro uso e após o seu reprocessamento (cada uso), indicando o número de usos do mesmo, bem como a data e assinatura do profissional responsável pelo reprocessamento.	0	1	2	3
6 - Troca de luvas a cada novo procedimento (como curativos e manuseio do sistema de hemodiálise) pelos profissionais de saúde.	0	1	2	3
**Pontuação Parcial:**				
Aspectos de não conformidade:				
**Processos Organizacionais de Gestão em Saúde**				
	NA	NC	PC	C
7 - Uso de etiquetas para registro da administração de medicamentos endovenosos com nome da medicação, dose, data, hora da diluição/aspiração, nome do profissional responsável e nome completo do paciente.	0	1	2	3
8 - Acondicionamento dos medicamentos potencialmente perigosos em local exclusivo, identificado, de acesso restrito e com controle de abertura.	0	1	2	3
9 - Utilização de isolador descartável para medir pressão arterial e venosa na máquina.	0	1	2	3
10 - Registro semestral da limpeza do reservatório de água potável, com assinatura do profissional responsável.	0	1	2	3
11 - Presença de posto de enfermagem próximo à sala de hemodiálise com facilidade de acesso e de visualização de todos os pacientes e com disponibilidade de recursos materiais necessários à assistência.	0	1	2	3
**Pontuação Parcial:**				
Aspectos de não conformidade:				
**Práticas Assistenciais para Controle de Infecção**				
	NA	NC	PC	C
12 - Retirada de adornos e utilização de EPIs pelos profissionais de saúde.	0	1	2	3
13 - Higienização das mãos, pelos profissionais de saúde, antes de tocar o paciente, antes de realizar procedimentos assépticos, após exposição a fluidos corpóreos e após contato com superfícies próximas ao paciente.	0	1	2	3
14 - Realização de curativos de cateteres e/ou fístulas, utilizando técnica asséptica.	0	1	2	3
15 - Realização de limpeza e secagem do chão a cada sessão de hemodiálise e sempre que necessário.	0	1	2	3
**Pontuação Parcial:**				
Aspectos de não conformidade:				
Pontuação Total:				

*Notas: 0- Não se aplica: todo o conteúdo do item não se aplica à realidade que está sendo avaliada ou não é possível ser observada. 1- Não Conforme: todo o conteúdo do item encontra-se em não conformidade com a realidade que está sendo avaliada. 2- Parcialmente conforme: apenas parte do conteúdo do item é condizente com a realidade que está sendo avaliada. 3- Conforme: todo o conteúdo do item encontra-se em conformidade com a realidade que está sendo avaliada.*

## DISCUSSÃO

Durante a validade da estrutura interna da EASPRCH, alguns itens foram excluídos devido ao desvio padrão igual a zero. Atribuem-se essas invariabilidades aos requisitos básicos exigidos para o funcionamento dos serviços de diálise e a outras normativas sobre segurança do paciente^(13,21)^.

Quanto à consistência interna, a EASPRCH apresentou valores de alfa de Cronbach semelhantes aos de outros instrumentos relacionados à segurança do paciente, como a Escala de Predisposição à Ocorrência de Eventos Adversos (EPEA) (Cronbach = 0,74 para a dimensão Estrutura e 0,93 para Processo)^(22)^, o *Safety Attitudes Questionnaire* (SAQ) (Cronbach = 0,89) e o *Hospital Survey on Patient Safety Culture* (HSOPSC) (Cronbach = 0,91)^(23,24)^.

A versão modificada da EASPRCH, com 15 itens, atingiu 72,41% de variância explicada, aproximando-se de outros instrumentos, como a Escala de Eventos Adversos Associados às Práticas de Enfermagem, que explicou 70,79% de variância com onze fatores^(25)^.

A matriz de componentes principais, com rotação Varimax, mostrou melhor divisão da escala em três fatores, a saber: (1) Procedimentos Estratégicos para Prevenção de Erros, com itens relacionados à identificação, desinfecção, funcionalidade e acondicionamento adequados dos insumos utilizados durante as sessões de hemodiálise, além do uso de equipamentos de proteção individual (EPIs); (2) Processos Organizacionais de Gestão em Saúde, readequado com itens como segurança na medicação, uso de isoladores descartáveis, normas e rotinas para tratamento da água e estrutura física funcional; e (3) Práticas Assistenciais para Controle de Infecção, incluindo normas e rotinas sobre o uso de EPIs, higienização das mãos, técnicas assépticas na realização de curativos e limpeza do ambiente.

Os itens que compõem as dimensões da escala demonstram que os eventos adversos decorrem da combinação de múltiplos fatores, incluindo características individuais dos pacientes, as condições dos profissionais de saúde e as variáveis institucionais. No setor de hemodiálise, essa complexidade é intensificada pelo uso de equipamentos avançados, pela necessidade de tratamento rigoroso da água, pela alta rotatividade dos pacientes e pela administração de medicamentos potencialmente perigosos. Além disso, o risco de infecção é elevado, exigindo cuidados meticulosos e protocolos de segurança bem definidos para minimizar complicações. Esses fatores combinados tornam o ambiente de hemodiálise particularmente suscetível a eventos adversos, destacando a importância de instrumentos que avaliem a segurança do paciente^(26)^.

As dimensões da EASPRCH estão associadas às seis áreas que a *Renal Physicians Association* (RPA) propõe para a melhoria da segurança do paciente (higienização das mãos; uso correto do dialisador e da solução de diálise; administração segura de medicamentos; seguimento de protocolos, normas e rotinas; prevenção de quedas; e cuidados relacionados à punção da fístula)^(9)^.

De acordo com um estudo de revisão, a análise dos itens e dos respectivos domínios da EASPRCH revela que estes estão alinhados com os fatores predisponentes, incapacitantes, precipitantes e reforçadores relacionados à cultura de segurança de pacientes renais crônicos em tratamento hemodialítico^(27)^. Um estudo desenvolvido no Japão, por exemplo, criou um sistema denominado *Taxonomia de Erro* e concluiu que mais de 70% dos incidentes de hemodiálise estavam associados a problemas ou complicações envolvendo o dialisador, o circuito ou a medicação. Esses achados reforçam a relevância das duas primeiras dimensões da escala, que abordam diretamente esses aspectos cruciais para a segurança do paciente^(28)^.

Já no México, concluiu-se que os eventos adversos mais prevalentes em pacientes submetidos à hemodiálise estão relacionados à infecção de acesso vascular, reforçando a importância da terceira dimensão da EASPRCH^(29)^.

Ademais, ao analisar a estabilidade temporal da escala, percebeu-se que o conteúdo dos itens que obtiveram Kappa abaixo do recomendado estava relacionado a fatores comportamentais dos profissionais, fato que, neste estudo, justifica a instabilidade temporal desses itens e a possibilidade de interferência externa. Estudos afirmam que valores mais baixos de Kappa estão associados à compreensão dos pesquisadores ou ao tempo entre as medidas, pois um intervalo de tempo excessivamente longo pode fazer com que o sujeito tenha alterado seu estado de saúde, modificando o objeto de avaliação^(30-32)^.

Finalmente, na parametrização, o ponto de corte da EASPRCH foi definido por meio da Curva ROC. Pressupõe-se que, quando a escala apresentar altos escores (>34), a ocorrência de incidentes será reduzida e, em contrapartida, escores baixos na escala (<34) poderão prever uma maior incidência de incidentes na hemodiálise.

A análise dos escores da escala, assim como de seus itens individualmente, pode contribuir para o gerenciamento de risco na ocorrência de incidentes durante as sessões de hemodiálise, dado que esse é um ambiente dinâmico e complexo. O gerenciamento de risco envolve um processo sistemático de identificação, análise e controle de riscos reais e potenciais, levando em conta as implicações associadas aos recursos disponíveis. Esse processo vai além da simples identificação de erros, destacando a importância de propor soluções proativas para reduzir e mitigar falhas, promovendo a segurança do paciente de forma contínua^(33)^.

Os pacientes hemodialíticos apresentam maior propensão a desenvolver distúrbios metabólicos, anormalidades de coagulação e infecções na corrente sanguínea. Esses problemas impactam diretamente o trabalho dos profissionais de saúde. Ressalta-se a importância do cuidado contínuo a esses indivíduos, com organização e planejamento voltados à prevenção e ao tratamento de complicações associadas ao procedimento^(34,35)^.

Logo, a aplicação e a análise das pontuações e das informações coletadas pela EASPRCH fornecerão dados para nortear e monitorar fragilidades funcionais e organizacionais, auxiliando na melhor capacitação dos profissionais e no aprimoramento do processo de trabalho da equipe de enfermagem.

### Limitações do estudo

Existem limitações que precisam ser elucidadas, como a realização restrita da coleta de dados, que, apesar de ter sido conduzida com uma amostra significativa da população, ocorreu em apenas três clínicas de hemodiálise situadas no mesmo estado e cidade, em cenários bastante similares. Ademais, sugere-se a realização de uma nova coleta de dados para testar a reprodutibilidade da escala, porém, desta vez, aplicando apenas um instrumento por vez, a fim de analisá-la novamente e verificar a capacidade de replicação dos dados da escala por diferentes pesquisadores. Sugere-se, também, a adaptação da EASPRCH para a realidade dos pacientes renais agudos, que realizam diálise em hospitais, um cenário ainda pouco estudado e que apresenta diversas alterações no estado clínico do paciente, fator que compromete a segurança.

### Contribuições para a Área

Este artigo fortalece a importância de instrumentos que norteiem e fomentem a temática da segurança do paciente em clínicas de hemodiálise, servindo como ponto de partida para o desenvolvimento de mais instrumentos na realidade brasileira, que aliem a segurança do paciente ao procedimento hemodialítico. Além disso, evidencia a necessidade do desenvolvimento de estudos que tragam recursos para a atuação prática dos Núcleos de Segurança do Paciente, bem como para o levantamento de incidentes e eventos adversos em hemodiálise.

## CONCLUSÕES

Disponibiliza-se aos leitores a versão modificada da EASPRCH, com evidências de validade de construto, confiabilidade e estabilidade, incluindo um ponto de corte que possibilita identificar fragilidades ou potencialidades relacionadas a determinados indicadores, os quais podem impactar ou não a segurança no atendimento ao paciente em hemodiálise. Dessa forma, torna-se viável o direcionamento de medidas e intervenções que potencializem práticas seguras durante o atendimento ao paciente em hemodiálise.

## Data Availability

Não se aplica.
